# Patient Empowerment and Agency through Intrapartum Counseling and Education (PEAICE): A randomized controlled trial

**DOI:** 10.1002/pmf2.70458

**Published:** 2026-09-24

**Authors:** Samuel J. F. Melville, Lorraine Codding, Blake M. Lee, Mónica Rincón, Bharti Garg, Katherine Lyons, Fei Cai

**Affiliations:** ^1^ Department of Obstetrics and Gynecology Oregon Health and Science University Portland Oregon USA

**Keywords:** childbirth, induction, labor, patient education, patient satisfaction

## Abstract

**Introduction:**

As more patients decline evidence‐based care from their obstetric providers, effective and accessible antenatal education is essential in reversing this course. This study aimed to assess the effectiveness of a novel low‐cost physical intrapartum labor guide on personal perceptions of patient control, labor satisfaction, and obstetric/neonatal outcomes.

**Methods:**

This was a single‐institution, randomized controlled trial of patients with singleton pregnancies undergoing induction of labor. Participants were randomized at admission for induction to intrapartum guide versus standard of care counseling. Primary outcomes included personal perception of control and satisfaction during labor (assessed via the Labor Agentry Scale [LAS] and a six‐question satisfaction survey, administered ≤7 days postpartum). Secondary outcomes included prespecified obstetric and neonatal outcomes. Comparisons between groups were done using Chi‐square/Fisher's exact test or two‐sample *t*‐test/Wilcoxon rank‐sum test. Multiple linear regression analysis was then used to examine the association of intrapartum guide education with LAS scores and neonatal outcomes. Covariate adjustment was performed to improve statistical precision and to protect against residual chance imbalance.

**Results:**

A total of 216 patients were enrolled and randomized (108 per arm). Of those enrolled, 189 (87.5%) completed the postpartum survey ≤7 days postpartum and were thus included in the analysis. Compared to standard of care, patients in the intrapartum guide group had significantly higher mean LAS scores (58.36 [95% CI, 56.78–59.94] vs. 54.29 [95% CI, 52.45–56.13]; *p* = .001) and remained so when adjusting for race, ethnicity, age, insurance, and mode of delivery (*β* = 3.42; 95% CI, 1.19–5.65). Patients in the intrapartum guide group reported significantly higher birth satisfaction, knowledge of childbirth, and satisfaction with intrapartum education. Finally, neonates of individuals randomized to the intrapartum guide were less likely to receive assisted ventilation (12.5% vs. 23.7%; *p* = 0.046); however, this difference was not significant when controlling for mode of delivery (adjusted odds ratio [aOR], 0.49; 95% CI, 0.21–1.12).

**Conclusion:**

A novel, low‐cost intrapartum labor guide administered during induction of labor significantly increased patient‐perceived agency and satisfaction. This represents a promising strategy for improving patient adoption and fidelity of labor education regarding evidence for interventions that potentially reduce obstetric/neonatal risk. The findings of this study warrant further study and implementation in obstetric practices.

**Trial Registration:**

ClinicalTrials.gov identifier: NCT06787521

## 1 INTRODUCTION

Childbirth education has historically been neglected in modern patient care. Although the Sheppard–Towner Act of 1921 marked one of the first federal investments in childbirth education, it did not become more formally organized and widely adopted until the 1960s during the civil rights and feminist movements [1]. Patients facing barriers may be more likely to seek medical information outside of the traditional medical profession. A 2025 study found that 38% of adults under age 35 have disregarded a healthcare provider's medical advice in favor of advice seen on social media [2]. In this era of social media and artificial intelligence, healthcare providers face growing challenges in maintaining visibility and influence within childbirth education [3, 4, 5].

Upon closer examination, the empirically reviewed accuracy of information from such alternative sources comes into question. A 2024 study reviewed the most popular TikTok videos on labor augmentation strategies, collectively receiving over 19 million views. Among these sources, 27% were made by health professionals and the mean score was consistent with information that was correlative with “serious or extensive shortcomings” [6].

As a result, patients may present for induction of labor without evidence‐based information consistent with professional guidelines regarding labor augmentation and risk‐reducing interventions. These new circumstances emphasize a critical need for education on childbirth with high fidelity (aligned with evidence‐based professional recommendations) and high adoption (decreased barriers to access including time and cost).

Furthermore, existing literature demonstrates that antenatal education administered prior to labor provides significant benefits for patients during the labor process [6, 7, 8]. These benefits are extremely heterogenous, consistent with the diversity of educational modality, techniques, and access. Studies have shown improvements in psychological outcomes [9, 10, 11] and labor satisfaction [12, 13, 14]. In addition to improved patient experience, antenatal education has also been shown to increase patient adherence to medically indicated induction/augmentation of labor across several studies [6, 9, 14, 15, 16]. Those who receive this education also have improvements in maternal outcomes, which may in part be due to adherence to evidence‐based care, though the effects across studies remain heterogeneous.

While these studies are promising, all antenatal education delivered outside the hospital setting imposes either a financial burden or an opportunity cost on the patient, which remains a significant reason for seeking alternative means of education. Furthermore, most studies’ methodologies did not present the scientific evidence behind the recommended interventions in labor to the patient. To our knowledge, no study has provided patients with access to physician‐facing information about the labor process. Therefore, the primary aim of this study is to evaluate the effectiveness of a low‐cost, evidence‐based, intrapartum labor guide to evaluate its impact on patient‐perceived agency in labor.

## 2 MATERIALS AND METHODS

### 2.1 Recruitment and Randomization

This was a randomized controlled trial conducted at a single, large academic medical center in Portland, Oregon. Participants were recruited between April 2025 and February 2026. Inclusion criteria included English‐speaking patients between ages 18 and 50 undergoing medical or elective induction of labor for singleton pregnancies >36 weeks of gestation. Patients excluded from the trial included those undergoing induction terminations or who had received a diagnosis of fetal demise or major fetal anomaly. Minors and adults with impaired decision‐making capacity were also excluded from the trial because these populations were anticipated to demonstrate greater variability in factors influencing information uptake, including education and level of comprehension. In addition, prospective enrollees who were approached at admission after the onset of active labor as defined by three regular contractions in 10 min or >5 cm dilation were also excluded to standardize patients.

The research team approached potential participants with an Institutional Review Board (IRB)‐approved introduction to the study, and participants consented to participate at that time. As part of the consent process, participants were told they would be given a “new counseling strategy” versus “standard of care,” but otherwise study participants were blinded. Both groups of patients received information about induction/labor in their clinics at the discretion of their prenatal care providers. After consent was obtained, participants were randomized using our institution's REDCap (Research Electronic Data Capture) in a 1:1 fashion. Participants randomized to the intrapartum guide were given the intrapartum labor guide in addition to standard of care counseling, while those randomized to the control received only “standard of care” verbal education deemed appropriate by providers’ clinical practice. There was no standardization of this education across practices. The obstetric team was notified of the patient's participation in a progress note and in the problem list of the electronic medical record (EMR). Clinicians were not informed of group assignment nor was group assignment discoverable via the medical record; however, true double blinding was impossible as providers may have learned of group assignment by visualizing the guide among patient possessions in the room, or by recognition based on the questions asked by the patient.

### 2.2 Intrapartum Labor Guide

Author S.J.F.M. wrote the intrapartum labor guide (see Supporting Information 1) with review from authors K.L. and F.C. A graphic designer was employed to make the guide visually appealing but did not have editorial discretion on the content of the guide. The guide was developed exclusively using recommendations and supporting evidence from the ACOG Clinical Practice Guideline Number 8, *First and Second Stage Labor Management* [17]. The nine‐page guide was organized into an introduction, stating the goals of labor management (vaginal delivery and reducing health risks for patient and neonate), followed by an explanation of the indications for induction of labor. After the introduction, the guide provided evidence for interventions broken into the following categories when they may be used in induction/augmentation: (1) cervical ripening, (2) early/latent labor, (3) active labor, (4) second stage of labor, and (5) QR code in which participants could view the Clinical Practice Guideline Number 8 in full. Following construction of the guide, readability was verified using the Flesch‐Kincaid Grade Level tool, ensuring the guide was written at or less than the eighth‐grade level [18].

### 2.3 Study Outcomes

The primary outcome, perceived control in labor, was assessed via the Labor Agentry Scale (LAS‐10), a validated 10‐questions tool to evaluate personal control during childbirth with a maximum score of 70 (Supporting Information 2). In addition to the LAS‐10, an additional six‐question survey with a minimum score of 0 to maximum score of 36 (Supporting Information 3) was administered to ascertain information on patient attitudes on the guide itself and satisfaction with the birth experience, adapted from the Perceived Control in Childbirth Scale (PCCh) [19]. Secondary outcomes included maternal uptake of interventions and key maternal/neonatal outcomes to investigate downstream effects of the labor guide. Maternal interventions/outcomes included mode of delivery, cervical ripening, balloon placement, misoprostol administration/dose number/total amount administered, oxytocin administration, intrauterine pressure catheter placement, manual rotation, category II tracing, category III tracing, and quantitative blood loss (QBL) at delivery. Neonatal outcomes included neonatal intensive care unit (NICU) admission, Apgar (appearance, pulse, grimace, activity, respiration) scores at 1 and 5 min (<7 & ≥7), assisted ventilation (any type), and seizure. Neonatal outcomes were chosen as a secondary outcome measure to support the implementation of the guide clinically at neonatal‐conscious institutions and to ensure that the interaction between short‐term neonatal outcomes and perceived control in labor was contextualized. Surveys with the LAS‐10 questions and the six supplemental satisfaction questions were distributed through REDCap, either in person using an iPad or via phone, 1–7 days postpartum (with postpartum Day 1 being defined as the 24 h beginning at midnight following the day of delivery). Patient demographics and obstetric and neonatal outcomes were collected via electronic medical record review after discharge from delivery.

### 2.4 Statistical Analysis

#### 2.4.1 Sample Size Calculation

Prior studies utilizing the LAS‐10 show average scores ranging between 50 and 57 out of 70, with standard deviations (SDs) ranging from 6 to 12 [15, 20, 21, 22]. The anticipated LAS‐10 score in the usual education group was 53.5 with SD of 6–12. Group sample size of 86 per arm was needed to achieve 81% power to detect a mean difference of 4 in LAS‐10 with an alpha of 0.05 and an SD of 9 for both groups based on 2000 Monte Carlo samples from the normal distribution [23, 24]. A mean difference of 4 (7.5% difference between intervention group and control group, relative to control group) was chosen as this was consistent with the mean % difference in LAS across the sample in several trials that tested low‐cost education intervention strategies such as podcasts (7.5%) [15], female family member presence (11.8%) [20], continuous intrapartum professional support (2.5%) [25], and perfect adherence to a Lamaze education program (9.91%) [25]. The chosen mean difference in LAS was also comparable to differences in LAS measured across demographics such as body mass index (BMI) > 30 (5.5%) [26], smoking status (3.1%) [27], African American race (3.83%) [27], Hispanic race (5.5%) [27], Asian race (5.4%) [27], government versus private insurance (5.1%) [27]; as well as across interventions such as operative delivery (2.5%) [27], cesarean section (9.2%) [27], and hands/knees pushing in second stage (1.1%) [28]. The percent difference in LAS was also comparable between the induction of labor group and expectant management group in the ARRIVE Trial (A Randomized Trial of Induction Versus Expectant Management) 6–92 h after delivery (2.4%) and 2–4 weeks from delivery (1.1%) [29]. This mean difference threshold or minimal clinically important difference (MCID) compares appropriately with relevant literature reviews of MCIDs chosen for RCTs [30]. Estimating a 20% loss to follow‐up rate, we planned to recruit 108 participants per arm or 216 total patients.

#### 2.4.2 Data Analysis

Analyses were performed as intention‐to‐treat. The privacy rights of all human subjects were strictly observed. Survey data were managed in REDCap, a secure, web‐based platform implemented to support data gathering and organization for research studies. Data collected were not always normally distributed, and thus comparisons among groups were done using Chi‐square/Fisher's exact test (categorical variables) and two‐sample *t*‐test/ Wilcoxon rank‐sum test (continuous variables, depending on the distribution of the data). Multivariable linear regression analysis was then utilized to assess the association of intrapartum guide education with LAS scores. No data transformation was done as LAS scores were normally distributed. For the neonatal outcomes, multivariable logistic regression analyses were performed to examine the association of intrapartum guide education with adverse neonatal outcomes (NICU admission, Apgar scores at 1 and 5 min [categorized to <7 and ≥7], and assisted ventilation), and adjusted odds ratio (aOR) with 95% confidence interval (CI) were reported. All regression models were adjusted for maternal race and ethnicity, parity, age, insurance, and mode of delivery. For the neonatal outcomes, multivariable Poisson regression analyses were performed to examine the association of intrapartum guide education with adverse neonatal outcomes (NICU admission, Apgar scores at 1 and 5 min, and assisted ventilation). Covariate adjustment was performed to improve statistical precision and to protect against residual chance imbalance, consistent with current FDA guidance on covariate adjustment in randomized trials [31]. Statistical significance was set at *p* < 0.05. Statistical analysis was performed using Stata, version 19 (StataCorp LLC). This study was approved by an IRB. Research personnel obtained written informed consent from all patients.

## 3 RESULTS

**FIGURE 1:**
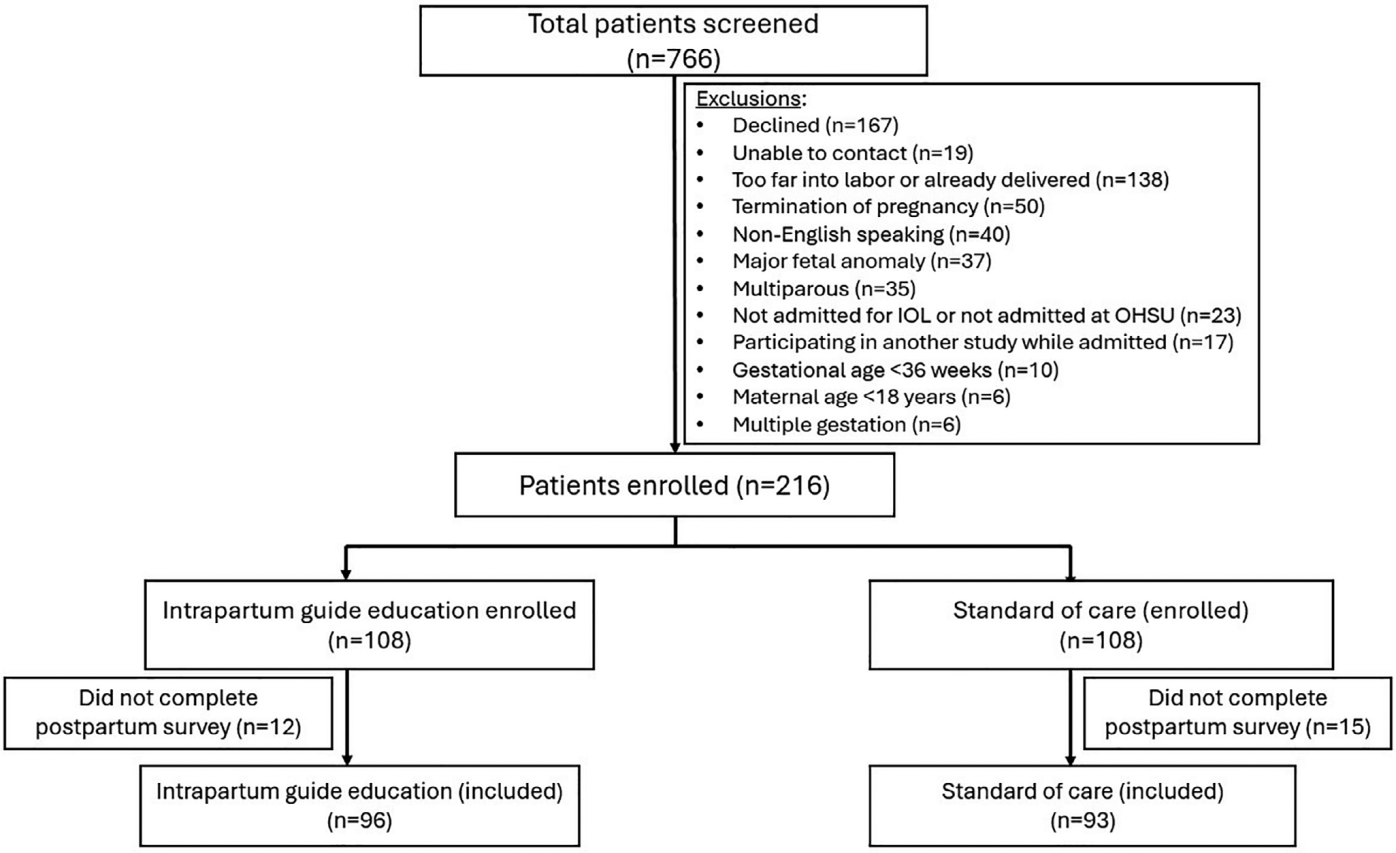
Flow diagram for PEAICE study participants.

Research personnel screened patients between April 2025 and February 2026. A total of 766 patients were screened for eligibility. Of those screened, 167 declined participation (21.8%), 19 were unable to be contacted due to logistic constraints (2.5%), and 362 met exclusion criteria (47.3%). Of the 216 enrolled and randomized, 12 patients in the intrapartum guide group and 15 patients in the control group were not reachable for the postpartum survey, including the primary outcome measure. Therefore, the final analysis included 189 patients (Figure 1).

Compared to controls, participants in the intrapartum guide group had significantly higher LAS‐10 scores (58.36 [95% CI, 56.78–59.94] vs. 54.29 [95% CI, 52.45–56.13]; *p* = 0.001). Multiple linear regression analysis showed that participants in the intrapartum guide group had higher LAS scores after adjusting for race, ethnicity, age, insurance, and mode of delivery (*β* = 3.42; 95% CI, 1.19–5.65).

There were no significant differences between groups in demographics, including maternal age, prepregnancy BMI, race or ethnic group, insurance status, gestational age at admission, diabetes status, hypertensive disease of pregnancy diagnosed prior to admission or during admission, smoking status, fetal growth restriction (diagnosed prior to admission), low birth weight, and type of induction (scheduled vs. unscheduled) (Table 1).

**TABLE 1:** Demographics of patients randomized to intrapartum guide education versus standard of care intrapartum counseling.

	Total *N* = 189	Standard of care *N* = 93	Intrapartum guide education *N* = 96
Age, mean ± SD	32.7 ± 4.9	33.3 ± 4.6	32.2 ± 5.2
Age, median (IQR)	33.0 (30.0–36.0)	33.0 (30.0–37.0)	33.0 (29.5–35.0)
Prepregnancy BMI			
<30	126 (66.7%)	62 (66.7%)	64 (66.7%)
30–40	48 (25.4%)	23 (24.7%)	25 (26.0%)
>40	14 (7.4%)	7 (7.5%)	7 (7.3%)
Missing	1 (0.5%)	1 (1.1%)	0 (0.0%)
Racial‐ethnicity			
Non‐Hispanic White	129 (68.3%)	67 (72.0%)	62 (64.6%)
Non‐Hispanic Black	5 (2.6%)	1 (1.1%)	4 (4.2%)
Hispanic	22 (11.6%)	7 (7.5%)	15 (15.6%)
Asian/Pacific‐Islander	16 (8.5%)	8 (8.6%)	8 (8.3%)
Other	15 (7.9%)	9 (9.7%)	6 (6.2%)
Decline	2 (1.1%)	1 (1.1%)	1 (1.0%)
Insurance			
Private	139 (73.5%)	69 (74.2%)	70 (72.9%)
Public	50 (26.5%)	24 (25.8%)	26 (27.1%)
Parity			
Nulliparous	132 (69.8%)	66 (71.0%)	66 (68.8%)
Multiparous	57 (30.2%)	27 (29.0%)	30 (31.2%)
Gestational age (weeks), mean ± SD	39.0 ± 1.1	39.1 ± 1.1	39.0 ± 1.1
Gestational age (weeks), median (IQR)	39.0 (38.7–39.7)	39.1 (39.0–39.9)	39.0 (38.6–39.7)
Diabetes status			
Type 1 DM	6 (3.2%)	3 (3.2%)	3 (3.1%)
Type 2 DM	1 (0.5%)	0 (0.0%)	1 (1.0%)
GDM	48 (25.4%)	23 (24.7%)	25 (26.0%)
Hypertensive disease of pregnancy			
Chronic hypertension	13 (6.9%)	7 (7.5%)	6 (6.2%)
Gestational hypertension	21 (11.1%)	9 (9.7%)	12 (12.5%)
Preeclampsia	16 (8.5%)	6 (6.5%)	10 (10.4%)
Smoker (>1 cigarette/day)	7 (3.7%)	3 (3.2%)	4 (4.2%)
Fetal growth restriction (<10th percentile last US at admission)	9 (4.8%)	5 (5.4%)	4 (4.2%)
Low birth weight	5 (2.6%)	4 (4.3%)	1 (1.0%)
Type of induction			
Unscheduled induction	41 (21.7%)	19 (20.4%)	22 (22.9%)
Scheduled induction	148 (78.3%)	74 (79.6%)	74 (77.1%)

*Note*: *N* (%) reported unless otherwise specified.

Abbreviations: BMI, body mass index; DM, diabetes mellitus; GDM, gestational diabetes mellitus; IQR, interquartile range; SD, standard deviation.

Between groups, there were minimal differences in labor interventions received (Table 2). More patients in the intrapartum guide group received misoprostol (88.5% vs. 75.3%; *p* = 0.02); however, the total number of doses (2.3 ± 1.6 vs. 2.2 ± 1.9; *p* = 0.68) and the total dosage given (198.5 ± 141.3 vs. 220.0 ± 146.2; *p* = 0.36) during admission were not different.

**TABLE 2:** Labor and delivery outcomes of patients randomized to intrapartum guide education versus standard of care intrapartum counseling.

	Total	Standard of care	Intrapartum guide education	
	*N* = 189	*N* = 93	*N* = 96	*p* ^a^
Mode of delivery				0.27
Vaginal	137 (72.5%)	64 (68.8%)	73 (76.0%)	
Cesarean	52 (27.5%)	29 (31.2%)	23 (24.0%)	
Cook balloon	136 (72.0%)	66 (71.0%)	70 (72.9%)	0.77
Misoprostol	155 (82.0%)	70 (75.3%)	85 (88.5%)	0.02
Misoprostol—Total number of times it was given (either oral or vaginal), mean ± SD	2.2 ± 1.8	2.2 ± 1.9	2.3 ± 1.6	0.68
Misoprostol total dose (total mg given in labor course during admission), mean ± SD	208.2 ± 143.5	220.0 ± 146.2	198. ± 141.3	0.36
Pitocin given	166 (87.8%)	81 (87.1%)	85 (88.5%)	0.76
IUPC	61 (32.3%)	30 (32.3%)	31 (32.3%)	1.00
Manual rotation	8 (4.2%)	5 (5.4%)	3 (3.1%)	0.44
Category II fetal heart tracing	141 (74.6%)	71 (76.3%)	70 (72.9%)	0.59
Category III fetal heart tracing	2 (1.1%)	0 (0.0%)	2 (2.1%)	0.16
QBL, mean ± SD	717.7 ± 587.1	757.9 ± 584.0	678.9 ± 590.5	0.36

*Note*: *N* (%) reported unless otherwise specified.

Abbreviations: IUPC, intrauterine pressure catheter; QBL, quantitative blood loss.

^a^Chi‐square/Fisher's exact test/two‐sample *t*‐test.

More patients in the intrapartum guide group accepted cook balloon placement, misoprostol, oxytocin administration, intrauterine pressure catheter placement, and manual rotation; however, this study was not sufficiently powered to detect significant differences in the above maternal outcomes (Table 2). Vaginal delivery rate was higher in the intrapartum guide group, but the result was not statistically significant (Table 2). While mean QBL was lower in the intervention group by 70cc, this result was not statistically significant (Table 2).

**FIGURE 2:**
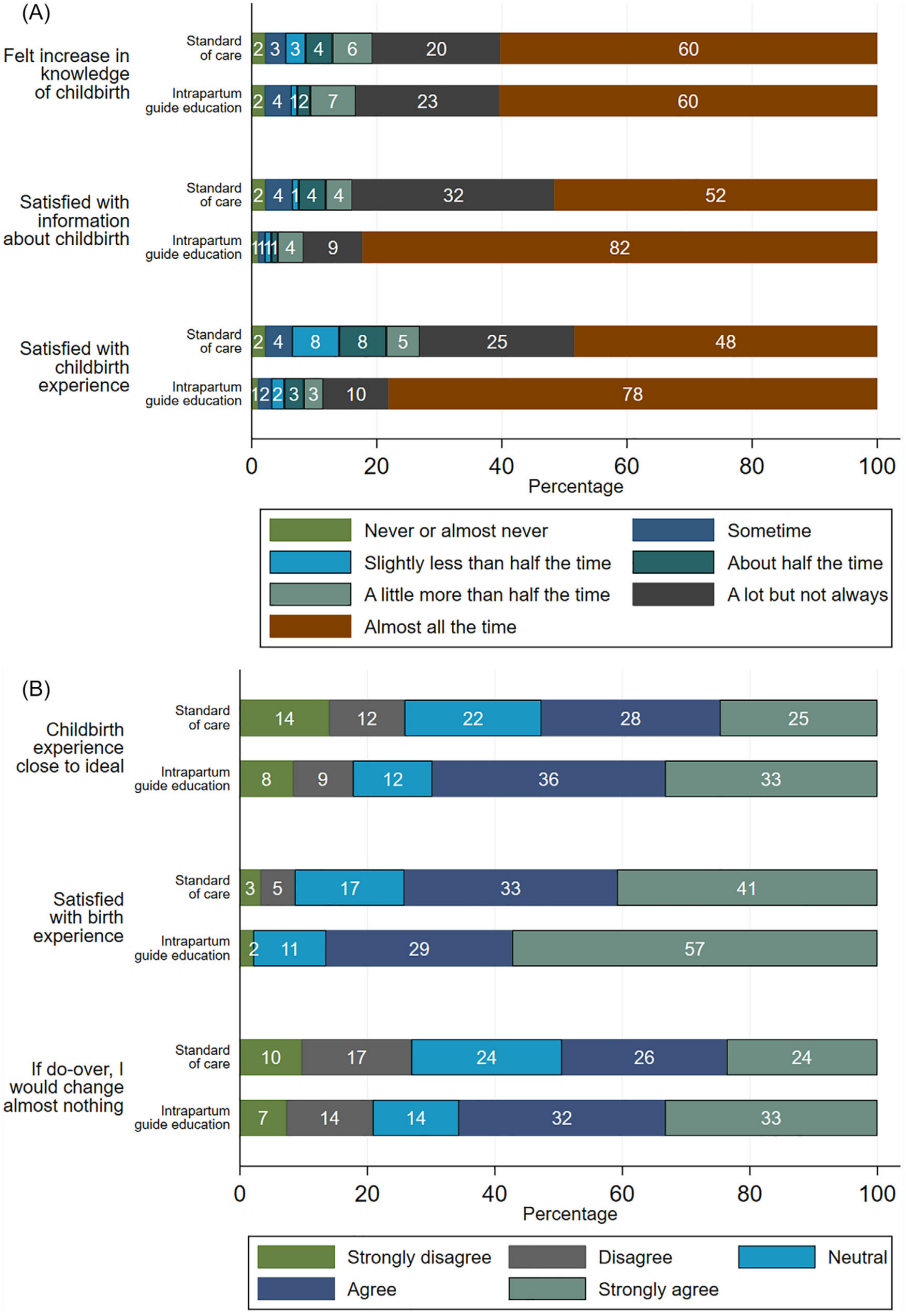
Labor satisfaction survey results.

Following delivery, there were no significant differences between groups in rates of NICU admission, Apgar scores at 1 and 5 min, or seizures. Neonates of patients who received intrapartum labor guide had lower rates of assisted ventilation postdelivery (12.5% vs. 23.7%; *p* = 0.046) (Table 3). This difference was not significant after adjusting for race and ethnicity, age, insurance, and mode of delivery (aOR, 0.49; 95% CI, 0.21–1.12) (Table 3). Compared to controls, participants in the intervention group reported significantly higher satisfaction with their baby's birth (5 [IQR 4–5] vs. 4 [IQR 3–5]; *p* = 0.01), increased knowledge of childbirth (7 [IQR 7–7] vs. 7 [IQR 5–7]; *p* < 0.001), satisfaction with information given to them on the birth experience (7 [IQR 7–7] vs. 7 [IQR 6–7]; *p* < 0.001), and that their childbirth experience was close to ideal (4 [IQR 3–5] vs. 4 [IQR 2–4]; *p* = 0.035). There were no significant differences between the intervention and control groups when participants were asked about satisfaction in the childbirth experience overall (7 [IQR 6–7] vs. 7 [IQR 6–7; *p* = 0.86) or if they would change anything about the experience in a do‐over (4 [IQR 3–5] vs. 3 [IQR 2–4]; *p* = 0.052) (Table 4). These findings are represented by answer responses in Figure 2A,B.

**TABLE 3:** Neonatal outcomes of patients randomized to intrapartum guide education versus standard of care intrapartum counseling.

	Total	Standard of care	Intrapartum guide education		
	*N* = 189	*N* = 93	*N* = 96	*p* ^a^	aOR (95% CI)^b^
NICU admission	21 (11.1%)	12 (12.9%)	9 (9.4%)	0.44	0.74 (0.28–1.94)
Apgar score at 1 min (<7)	33 (17.5%)	21 (22.6%)	12 (12.5%)	0.07	0.56 (0.24–1.31)
Apgar score at 5 min (<7)	8 (4.2%)	3 (3.2%)	5 (5.2%)	0.48	2.04 (0.39–10.59)
Assisted ventilation (any type)	34 (18.0%)	22 (23.7%)	12 (12.5%)	0.046	0.51 (0.21–1.19)
Seizures	1 (0.5%)	1 (1.1%)	0 (0.0%)	0.31	Not enough sample size

*Note*: *N* (%) reported unless otherwise specified.

Abbreviations: aOR, adjusted odds ratio; Apgar, appearance, pulse, grimace, activity, respiration; CI, confidence interval; NICU: neonatal intensive care unit.

^a^ Chi‐square/Fisher's exact test.

^b^ Adjusted for race and ethnicity, age, insurance, parity, and mode of delivery.

**TABLE 4:** Birth satisfaction survey outcomes of patients randomized to intrapartum guide education versus standard of care intrapartum counseling.

	Total	Standard of care	Intrapartum guide education	
	*N* = 189	*N* = 93	*N* = 96	*p* *
LAS scores, mean ± SD; Max 70	56.36 ± 8.61	54.29 ± 8.94	58.36 ± 7.81	0.001
Satisfied with childbirth experience overall, median (IQR); Max 7	7 (6–7)	7 (6–7)	7 (6–7)	0.86
Satisfied with information about birth experience, median (IQR); Max 7	7 (6–7)	7 (6–7)	7 (7–7)	<0.001
Felt increase in knowledge of childbirth, median (IQR); Max 7	7 (6–7)	6 (5–7)	7 (7–7)	<0.001
Childbirth experience close to ideal, median (IQR); Max 5	4 (3–5)	4 (2–4)	4 (3–5)	0.035
Satisfied with experience of my baby's birth, median (IQR); Max 5	4 (4–5)	4 (3–5)	5 (4–5)	0.01
If do‐over, I would change almost nothing, median (IQR); Max 5	4 (3–5)	3 (2–4)	4 (3–5)	0.052

*Note*: The components of the survey were on the Likert scale. Comparisons were done using the Wilcoxon rank‐sum test/two‐sample *t*‐test.

Abbreviations: aOR, adjusted odds ratio; CI, confidence interval; IQR, interquartile range; LAS, Labor Agentry Scale; SD, standard deviation.

* Chi‐square/Fisher's exact test/two‐sample *t*‐test.

## 4 DISCUSSION

This randomized controlled trial of patients undergoing induction of labor demonstrated that those who received a low‐cost evidence‐based intrapartum labor guide experienced significantly higher agency in labor (LAS‐10 scores), controlling for race, ethnicity, age, insurance, and mode of delivery. The mean difference in LAS‐10 between the intrapartum guide group and the control group (7.5%) was comparable to differences in LAS based on low‐cost interventions [15, 20], high‐cost interventions [25], mode of delivery [26], BMI [26], smoking status [26], and induction versus spontaneous labor [26, 28]. Unlike the trial that evaluated another low‐cost, labor education intervention that cost patients minimal time and effort (podcasts) [15], the difference in LAS‐10 between intervention and control in this trial was statistically significant. Our findings also support a favorable comparison between a low‐cost intrapartum education strategy that increased patient satisfaction regarding their education and birth, compared to studies of pre‐admission strategies demonstrating increased labor satisfaction albeit at higher cost and decreased access [13, 14, 25, 31].

There is a paucity of literature for intrapartum education performed at the time of admission; however, when compared to a randomized control trial of a similar, low‐cost, asynchronous form of labor education (podcasts) available prior to and during the birthing process, our study was consistent with findings of increased satisfaction but was also able to demonstrate increased perceived agency in the labor process [15]. It is possible this difference in perceived agency reflects the recency and physical nature of the education provided in our study, increased presentation of evidence for the labor interventions covered in the educational materials, or higher adoption/fidelity of the educational materials due to their recency and reduced barriers to access.

As our study was performed on individuals admitted for labor induction, we did not measure rates of acceptance of augmentation as patients not accepting augmentation during an admission for induction typically leave the hospital and would have been excluded from this trial. Therefore, this study is unable to validate prior literature indicating increased acceptance of augmentation when antenatal education is provided [6, 9, 14, 15, 16]. In this study, more individuals in the intrapartum guide group accepted misoprostol, cervical ripening balloon, and oxytocin than individuals in the control group; however, based upon the results, this study was not adequately powered to find statistically significant differences in augmentation/induction tool acceptance.

With regard to obstetric outcomes, prior studies have reported higher vaginal delivery rates in groups receiving childbirth education, but were unable to report differences in QBL [6, 9, 16, 32, 33, 34, 35, 36]. Our study did not find differences in mode of delivery or estimated blood loss. The rate of cesarean section and mean QBL was lower in the intrapartum guide group, but these findings were not statistically significant and thus cannot be reported as true differences.

In contrast to prior studies focused on the benefits of antenatal education literature for patients [13, 14, 37, 38], this study found significant differences in a neonatal outcome (assisted ventilation). These differences were not significant when adjusted for mode of delivery. While accepting more risk‐reducing interventions may have decreased the cesarean rate in the intrapartum guide group, thus decreasing the neonatal morbidity associated with cesarean section, it is also possible that the higher cesarean delivery rate in the intrapartum guide group was due to random chance (*p* = 0.27). This randomized controlled trial evaluated the impact of a low‐cost labor education strategy designed to reduce barriers to information and improve the availability of evidence‐based resources for patients. This method represents a strong contrast to social media, AI, and other previously mentioned modalities that patients are turning toward. In previous studies on pre‐admission classes, barriers to care remain high and there exist disparities in class attendance between patient demographic groups [39, 40]. An intervention aimed specifically during admission in theory should reduce those barriers. Furthermore, the inclusion of evidence‐based summaries and links to provider‐facing data within the guide was intended to reduce the paternalistic approach historically present in antenatal education, in which obstetric providers recommend interventions without directly sharing the supporting evidence with patients. It is possible that these specific innovations contributed to the statistically significant differences between the intrapartum guide and standard of care groups.

The strength of this study lies in the fact that it is a large randomized controlled trial that was able to capture individuals who may have had barriers to receiving costly modalities of antenatal education either requiring digital access or pre‐admission time opportunity cost. Blinding the participants from group assignment significantly reduced potential performance/participant bias. The resources utilized to recruit patients 24 h per day, 7 days per week led to adequate recruitment of patients to meet the power calculations and provide a true representative population of inductions at a tertiary care center.

This study had several limitations, including the potential for selection bias because only one quarter of patients approached were consented, enrolled, and completed the follow‐up questionnaires. It is possible that those individuals who did not consent or were lost to follow‐up had unmeasured differences from the target population. Limitations in follow‐up may have contributed to the loss of individuals in the study with unmeasured differences with the target population. Negation of this limitation was attempted through survey distribution by research personnel via direct telephone or in‐person contact on more than one attempt. In addition, we did not evaluate other factors that may have affected agency, satisfaction, or outcomes, including additional labor education materials, number of prenatal visits, and pre‐existing education status. We were also unable to measure adherence to educational materials. If a patient receiving the labor guide promptly discarded it, they would not have experienced the full benefit of the intervention and thus their potential outcome differences may be understated. Outcome differences may also be understated due to the potential for obstetric provider awareness of the trial and thus modifications to counseling approach universally. Despite these limitations, we anticipate the randomized controlled trial approach minimized the measurable and unmeasurable confounders on the outcomes of interest. Lastly, this study was conducted at a single, large academic center and labor guides were only available in English. Care was taken to write the guide at an eighth‐grade reading level to capture as many as possible with low healthcare literacy, the broadly applicable administration (without the need for technology, clinic time/space, patient financial/opportunity cost), and the novel approach to labor counseling in which tangible empirical impact is cited as support for recommendation all support this study's generalizability. However, limitations to its generalizability include its single‐study design and resulting narrow demography of the study population. Future studies should therefore invest in increased participant accessibility through other languages and clinical settings to increase the generalizability of an evidence‐based supplemental written labor interventions guide. While cost‐prohibitive for this study, further research should be adequately powered to detect differences in obstetric and neonatal findings to further support clinical benefit. Finally, the ceiling effect of our measures of satisfaction may have contributed to underestimations of patient satisfaction in the intrapartum guide group compared to the control group, as average satisfaction was closer to the maximum score parameter in the intrapartum guide group across all satisfaction parameters.

## 5 CONCLUSIONS

In this study, we created a low‐cost, evidence‐based, intrapartum labor intervention education strategy that significantly increased patient‐perceived agency and satisfaction in the birth process. Given our study's findings, low‐cost intrapartum labor guides may be a promising and currently underutilized modality for supplementing pre‐existing antenatal education programs to increase patient‐perceived control and satisfaction, warranting further research and increased utilization.

## AUTHOR CONTRIBUTIONS

**Samuel J.F. Melville**: Conceptualization; writing—original draft preparation; writing—review and editing; investigation; methodology; project administration; resources; funding acquisition; formal analysis; data curation; visualization. **Blake M. Lee**: Investigation; methodology; data curation; writing—review and editing. **Lorraine Codding**: Investigation; data curation; methodology; writing—review and editing. **Mónica Rincón**: Investigation; methodology; writing—review and editing; supervision; data curation. **Bharti Garg**: Methodology; validation; visualization; writing—review and editing; software; data curation. **Katherine Lyons**: Conceptualization; writing—review and editing. **Fei Cai**: Investigation; methodology; writing—review and editing; supervision; data curation.

## CONFLICT OF INTEREST STATEMENT

The authors declare no conflicts of interest.

## ETHICS STATEMENT

This randomized controlled trial was approved by the Oregon Health and Science Institutional Review Board (IRB#00028141, OnCore ID: 9294, eCRIS ID: CRS00009192). All patients gave written informed consent for their participation.

## Supporting information



Supporting information

Supporting information

Supporting information

## Data Availability

The data that support the findings of this study are available on request from the corresponding author. The data are not publicly available due to privacy or ethical restrictions.
