# Supplementary material for: Patient Empowerment and Agency through Intrapartum Counseling and Education (PEAICE): A randomized controlled trial
Source: Pregnancy (Hoboken). 2026 Sep 24;2(5):e70458. doi: 10.1002/pmf2.70458 (PMC13601936; doi:10.1002/pmf2.70458)

EVIDENCE-BASED GUIDE TO LABOR MANAGEMENT

OUR GOAL

Labor-management strategies should aim to increase the chances of a vaginal birth and reduce health risks for both the patient and baby. At the same time, they should consider the patient's preferences and values. This can be achieved by working closely with healthcare providers and nursing staff to make decisions together (American College of Obstetricians and Gynecologists, 2024).

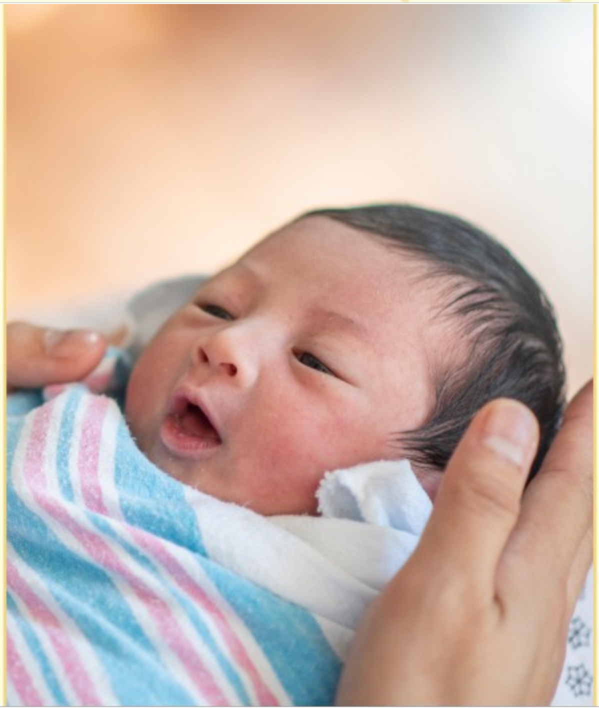

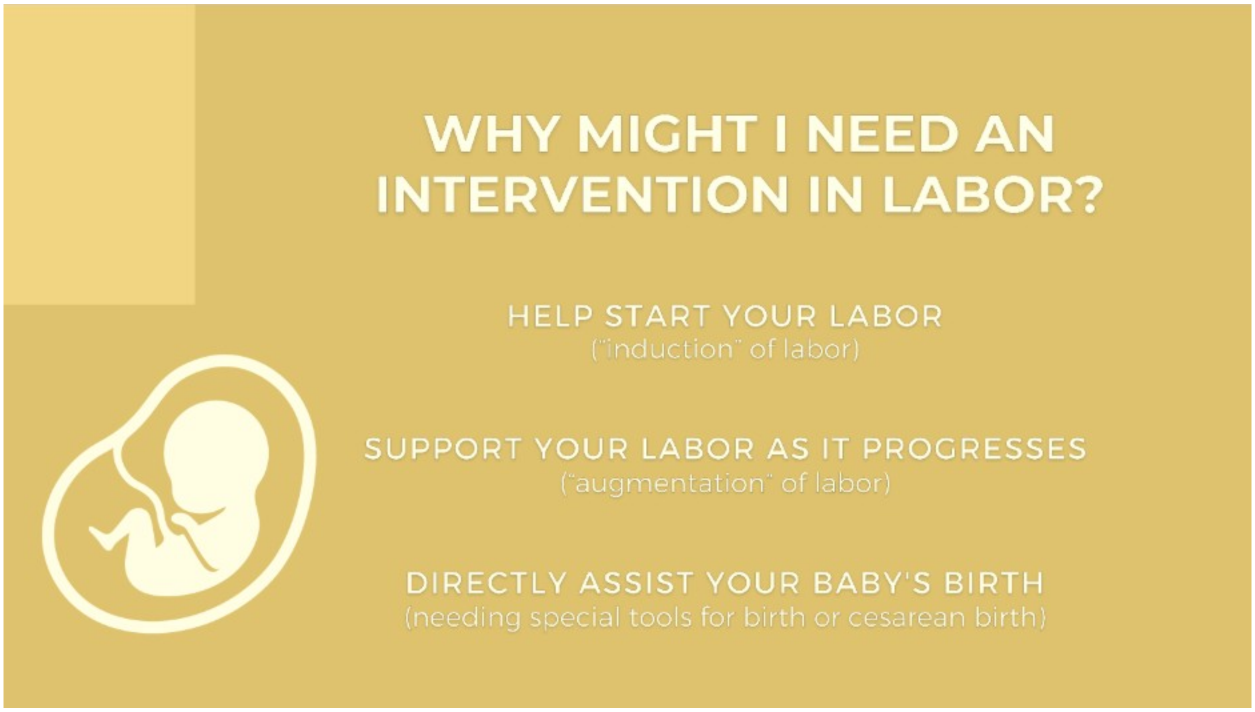
WHY MIGHT I NEED AN INTERVENTION IN LABOR?
HELP START YOUR LABOR
("induction" of labor)
SUPPORT YOUR LABOR AS IT PROGRESSES
("augmentation" of labor)
DIRECTLY ASSIST YOUR BABY'S BIRTH
(needing special tools for birth or cesarean birth)

THERE ARE MANY REASONS YOUR DOCTOR OR MIDWIFE MIGHT RECOMMEND AN INTERVENTION DURING LABOR, INCLUDING IF:

- you have a health condition that places you at higher risk for complications in labor
- your water broke but labor has not started
- your labor is progressing slowly and you make the decision with the team to augment your labor to reduce the possibility of c-section
- your baby is in a position that will make vaginal birth more difficult
- your baby is showing signs of distress via electronic fetal monitoring

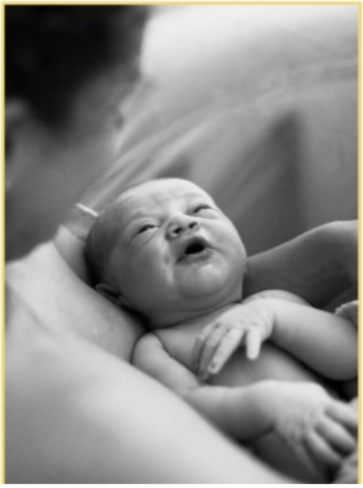

SUMMARY OF POTENTIAL INTERVENTIONS

One Method Only

Misoprostol
+ Balloon Catheter
(4-5 hours shorter)

- Labor Begins
- Vaginal Delivery

1. Cervical Ripening (you just arrived at the hospital and are not yet in labor)

a. **Misoprostol with balloon catheter:**

i. Studies have looked at how well different methods for starting labor work and how safe they are. In a recent collection of large studies, misoprostol combined with a balloon was found to be the best method. This combination gave the highest chance of having a vaginal delivery within 24 hours, lowered the chances of needing a C-section, and made vaginal delivery happen about 4-5 hours faster compared to one method alone. It also resulted in fewer problems, like needing to send the baby to the NICU, overly strong contractions, and changes in the baby's heart rate.

ii. Studies about patient satisfaction haven't shown that one cervical ripening method is preferred over the others. However, there are ways to manage pain during cervical checks and when placing the balloon catheter. These include IV pain medicine and a numbing cream.

b. **Cervical exams:** There is not enough evidence to determine the best frequency for exams during labor. Additionally, data from three large studies are mixed on whether the number of cervical exams is linked to a higher risk of fever during labor. Therefore, exams are done when necessary to monitor labor progress, since a longer labor can increase the risk of infection and bleeding after delivery.

SUMMARY OF POTENTIAL INTERVENTIONS

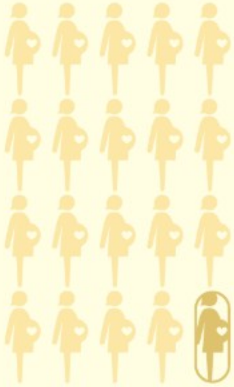

For every 20 patients who used oxytocin, 1 fewer patient needed a cesarean birth.

2. Early/Latent Labor (cervix is < 6 cm dilation)

a. **Epidural**: an epidural itself and the timing of the epidural does not increase the risk of cesarean birth.

b. **Early Amniotomy (breaking your water within 1 hour after the cervical balloon is removed)**: Several large studies have shown that the total time in labor is reduced by an average of 5 hours for first-time laboring patients without increasing the risk of cesarean birth, infection, bleeding, or pelvic floor trauma. At OHSU, cord prolapse occurs in only 1 in 1,000 full-term births, and there is no difference in the rate of cord prolapse whether the amniotomy is done by a provider or happens naturally.

c. **Oxytocin (Pitocin)**: During labor induction, this medication is used to start contractions once your cervix is 3-4 cm dilated. If labor slows down or stops, oxytocin is often used to help. Studies have shown that starting oxytocin when labor progress is slow can increase the chance of a spontaneous vaginal delivery. For every 20 patients who used oxytocin, 1 fewer patient needed a cesarean birth. Additionally, early use of oxytocin is linked to a reduced need for antibiotics during labor and a shorter labor overall.

SUMMARY OF POTENTIAL INTERVENTIONS

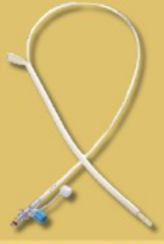

Intrauterine pressure catheters (IUPC)

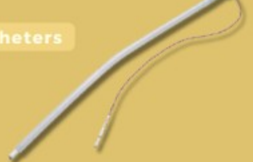

Fetal scalp electrodes (FSE)

3. Active Labor (cervix > 6 cm dilated)

a. **IUPC: (Intrauterine pressure catheters):** are often used after breaking the water if labor is slow or if it's hard to measure contractions accurately. Studies have shown that using an IUPC does not harm the laboring person or the baby.

b. FSE: (**Fetal scalp electrodes):** are used when external monitoring isn't giving enough information and there are concerns about how well your baby is handling labor. Large studies have found that using FSE can slightly reduce the need for forceps or vacuum deliveries, and it doesn't significantly increase the risk of injury to the baby's scalp.

SUMMARY OF POTENTIAL INTERVENTIONS

4. Second Stage of Labor (your cervix is 10 cm dilated and you have begun efforts to deliver)

a. **Manual rotation:** As delivery approaches, the baby's head might not be in the best position for birth—like if the baby is looking up instead of down. In such cases, your provider might suggest a manual rotation after you've been given enough anesthesia. Studies have shown that doing this can significantly lower the chance of needing a cesarean birth.

b. **Preventing tears:** warm compress, perineal massage, perineal support: A review of many large studies found that these interventions during the second stage of labor may reduce deeper tears (third and fourth degree) but did not reduce the overall chance of having a tear.

c. **Positioning:** Studies have found that whether a laboring person is on their hands and knees or lying on their side during labor doesn't affect the chances of needing special tools for delivery, having a cesarean birth, satisfaction with labor, the baby's condition at delivery, the need for extra care in the NICU, or perineal tears. However, if you find alternative positioning comfortable during labor, there is no medical reason not to use them!

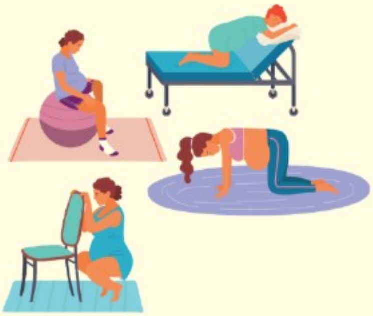

More detailed information on the labor intervention guidelines and the evidence supporting them can be found at:

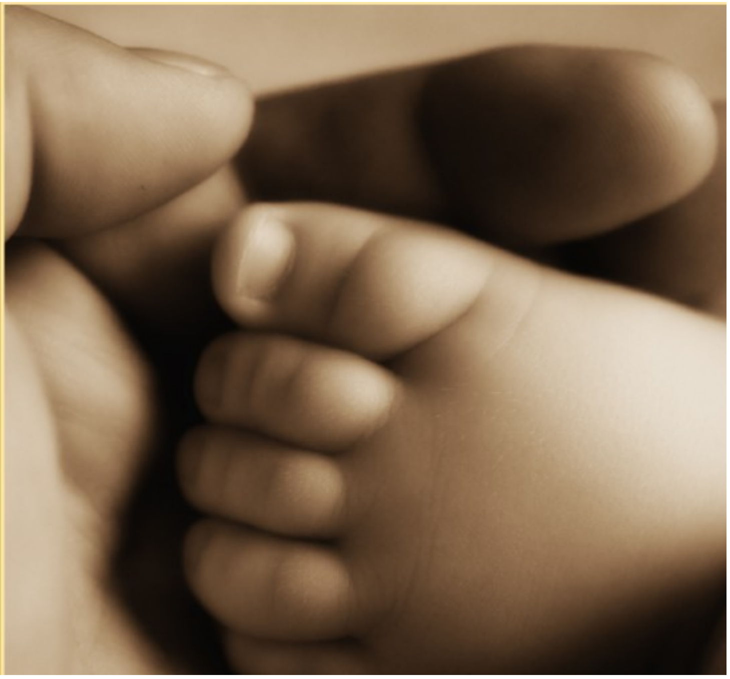

Supplement: Supplementary file 1 [file PMF2-2-e70458-s002.docx]
