# Supplementary material for: Patient Empowerment and Agency through Intrapartum Counseling and Education (PEAICE): A randomized controlled trial
Source: Pregnancy (Hoboken). 2026 Sep 24;2(5):e70458. doi: 10.1002/pmf2.70458 (PMC13601936; doi:10.1002/pmf2.70458)
Supplement: Supplementary file 1 [file PMF2-2-e70458-s001.docx]

**Postpartum Satisfaction Survey:**

I was satisfied with my childbirth experience overall

1. Almost all of the time
2. A lot but not always
3. A little more than half the time
4. About half the time
5. Slightly less than half the time
6. Sometimes
7. Never or almost never

I was satisfied with the information I received about childbirth

1. Almost all of the time
2. A lot but not always
3. A little more than half the time
4. About half the time
5. Slightly less than half the time
6. Sometimes
7. Never or almost never

I felt like I had increased knowledge about my childbirth experience

1. Almost all of the time
2. A lot but not always
3. A little more than half the time
4. About half the time
5. Slightly less than half the time
6. Sometimes
7. Never or almost never

In most ways, my childbirth experience was close to my ideal.

1. Strongly agree
2. Agree
3. Neutral
4. Disagree
5. Strongly disagree

I am satisfied with the experience of my baby’s birth.

1. Strongly agree
2. Agree
3. Neutral
4. Disagree
5. Strongly disagree

If I could do it over, I would change almost nothing about my childbirth experience.

1. Strongly agree
2. Agree
3. Neutral
4. Disagree
5. Strongly disagree
