# Supplementary material for: Patient Empowerment and Agency through Intrapartum Counseling and Education (PEAICE): A randomized controlled trial
Source: Pregnancy (Hoboken). 2026 Sep 24;2(5):e70458. doi: 10.1002/pmf2.70458 (PMC13601936; doi:10.1002/pmf2.70458)
Supplement: Supplementary file 3 [file PMF2-2-e70458-s003.docx]

LAS-10 Survey

Please complete the survey below. There is a second page to this survey. Thank you!

First Name: ______________

Last Name: ______________

Date of delivery: __________

**Please answer the following on a sale of 1-7 with your regards to your childbirth experience.**

**LAS10**

I felt tense

1. Almost all of the time
2. A lot but not always
3. A little more than half the time
4. About half the time
5. Slightly less than half the time
6. Sometimes
7. Never or almost never

I felt important

1. Almost all of the time
2. A lot but not always
3. A little more than half the time
4. About half the time
5. Slightly less than half the time
6. Sometimes
7. Never or almost never

I felt confidence

1. Almost all of the time
2. A lot but not always
3. A little more than half the time
4. About half the time
5. Slightly less than half the time
6. Sometimes
7. Never or almost never

I felt in control

1. Almost all of the time
2. A lot but not always
3. A little more than half the time
4. About half the time
5. Slightly less than half the time
6. Sometimes
7. Never or almost never

I felt fearful

1. Almost all of the time
2. A lot but not always
3. A little more than half the time
4. About half the time
5. Slightly less than half the time
6. Sometimes
7. Never or almost never

I felt relaxed

1. Almost all of the time
2. A lot but not always
3. A little more than half the time
4. About half the time
5. Slightly less than half the time
6. Sometimes
7. Never or almost never

I felt good about my behavior

1. Almost all of the time
2. A lot but not always
3. A little more than half the time
4. About half the time
5. Slightly less than half the time
6. Sometimes
7. Never or almost never

I felt helpless (powerless)

1. Almost all of the time
2. A lot but not always
3. A little more than half the time
4. About half the time
5. Slightly less than half the time
6. Sometimes
7. Never or almost never

I felt I was with people who cared about me

1. Almost all of the time
2. A lot but not always
3. A little more than half the time
4. About half the time
5. Slightly less than half the time
6. Sometimes
7. Never or almost never

I felt like a failure

1. Almost all of the time
2. A lot but not always
3. A little more than half the time
4. About half the time
5. Slightly less than half the time
6. Sometimes
7. Never or almost never
